# Detection and alterations of acetylcarnitine (AC) in human liver by ^1^H MRS at 3T after supplementation with l‐carnitine

**DOI:** 10.1002/mrm.29544

**Published:** 2022-12-27

**Authors:** Dragana Savic, Ferenc E. Mózes, Peregrine G. Green, Matthew K. Burrage, Mette Skalshøi Kjær, Leanne Hodson, Stefan Neubauer, Michael Pavlides, Ladislav Valkovič

**Affiliations:** ^1^ The Oxford Centre for Clinical Magnetic Resonance Research (OCMR), Radcliffe Department of Medicine University of Oxford Oxford UK; ^2^ Oxford Centre for Diabetes, Endocrinology and Metabolism University of Oxford Oxford UK; ^3^ Faculty of Medicine University of Queensland St Lucia Queensland Australia; ^4^ Novo Nordisk A/S Bagsvaerd Denmark; ^5^ Oxford NIHR Biomedical Research Centre University of Oxford Oxford UK; ^6^ Translational Gastroenterology Unit University of Oxford Oxford UK; ^7^ Department of Imaging Methods Institute of Measurement Science, Slovak Academy of Sciences Bratislava Slovakia

**Keywords:** acetylcarnitine measurements, acetylcarnitine, ^1^H‐MRS, carnitine supplementation, liver spectroscopy

## Abstract

**Purpose:**

Acetylcarnitine can be assessed in vivo using proton MRS (^1^H‐MRS) with long TEs and this has been previously applied successfully in muscle. The aim of this study was to evaluate a ^1^H‐MRS technique for liver acetylcarnitine quantification in healthy humans before and after l‐carnitine supplementation.

**Method:**

Baseline acetylcarnitine levels were quantified using a STEAM sequence with prolonged TE in 15 healthy adults. Using STEAM with four different TEs was evaluated in phantoms. To assess reproducibility of the measurements, five of the participants had repeated ^1^H‐MRS without receiving l‐carnitine supplementation. To determine if liver acetylcarnitine could be changed after l‐carnitine supplementation, acetylcarnitine was quantified 2 h after intravenous l‐carnitine supplementation (50 mg/kg body weight) in the other 10 participants. Hepatic lipids were also quantified from the ^1^H‐MRS spectra.

**Results:**

There was good separation between the acetylcarnitine and fat in the phantoms using TE = 100 ms. Hepatic acetylcarnitine levels were reproducible (coefficient of reproducibility = 0.049%) and there was a significant (*p* < 0.001) increase in the relative abundance after a single supplementation of l‐carnitine. Hepatic allylic, methyl, and methylene peaks were not altered by l‐carnitine supplementation in healthy volunteers.

**Conclusion:**

Our results demonstrate that our ^1^H‐MRS technique could be used to measure acetylcarnitine in the liver and detect changes following intravenous supplementation in healthy adults despite the presence of lipids. Our techniques should be explored further in the study of fatty liver disease, where acetylcarnitine is suggested to be altered due to hepatic inflexibilities.

## Abstract 2

Click here for author‐reader discussions

## 1 INTRODUCTION

**FIGURE 1:**
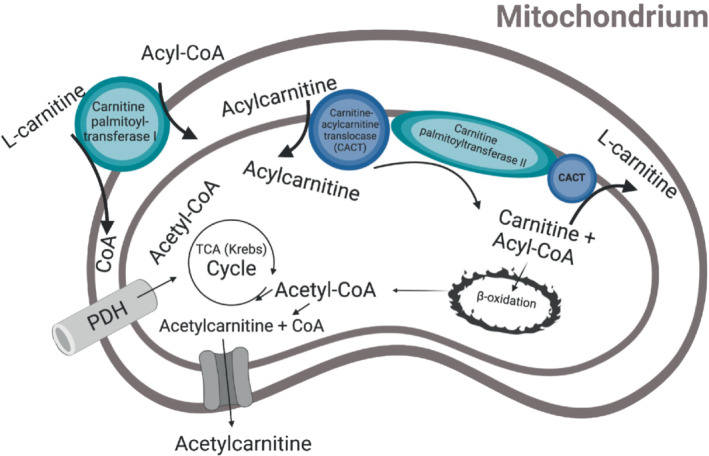
The l‐carnitine shuttle is illustrated in the mitochondrion. l‐carnitine binds to acyl‐CoA to be transported β‐oxidation, but it can also buffer acetyl‐CoA and thereby form acetylcarnitine. Figure adapted from Savic et al. (2020).^4^

l‐carnitine is required for the transportation of fatty acids into the mitochondria. It also modulates metabolic flexibility, by regulating the intracellular acetyl‐CoA/CoA ratio, which controls the balance between carbohydrate and lipid oxidation.^1^ Depending on the metabolic requirements of the cell, enzymes like carnitine palmitoyltransferase can convert l‐carnitine to acetylcarnitine which is a reversible reaction.^2^ Through the formation of acetylcarnitine, l‐carnitine sustains aerobic pyruvate oxidation because the high levels of acetyl‐CoA inhibit pyruvate dehydrogenase complex activity.^3^ The l‐carnitine shuttle is illustrated in Figure 1. The organs that are the main consumers of l‐carnitine, and therefore have the highest concentration of l‐carnitine species in the body are: heart (3500–6000 nmol/g tissue), skeletal muscle (2000–4600 nmol/g tissue), and the liver (1000–1900 nmol/g tissue).^5^ Carnitine species consist of free l‐carnitine and esterified carnitines, including acylcarnitines where acetylcarnitine under normal metabolic conditions, is the main source.^6^

Current methods to detect l‐carnitine or acylcarnitine species involve measuring it in blood, urine, or in tissue biopsies. Blood acylcarnitine levels do not always reflect the levels in specific tissues,^7^ and caution must be taken when analyzing blood results. Non‐invasive techniques using magnetic resonance spectroscopy and hyperpolarized MRI have emerged, and these allow for direct investigation of hepatic acetylcarnitine in vivo. Acetylcarnitine has been imaged with [2‐^13^C]pyruvate^8^ and [(1,2‐^13^C_2_)acetyl]‐l‐carnitine infusions^9^ in rats using hyperpolarized MRI. This however requires specialized equipment, and no groups to date have measured in vivo hepatic acetylcarnitine using ^1^H‐MRS. Skeletal muscle acetylcarnitine levels have been previously quantified using long TEs ^1^H‐MRS^10^, ^11^ where it was found that endurance athletes had higher levels of acetylcarnitine and lower levels of free l‐carnitine compared to non‐athletes.^7^ Skeletal muscle acetylcarnitine levels have also been reported to be lower in patients with type 2 diabetes compared to endurance athletes, and there was a positive correlation between muscle acetylcarnitine levels and insulin sensitivity.^11^ Based on these observations, it has been suggested that acetylcarnitine plays a role in maintaining metabolic flexibility and insulin sensitivity. Even though the muscle contains more than 75% of the total carnitine pool, it has low net fluxes of acetylcarnitine in both fasted and fed states and interacts very little with whole body metabolism,^4^ while the liver has a more key role in whole body acetylcarnitine metabolism.^12^ Given the liver, plays a key role in regulating systemic metabolic flexibility, investigating hepatic acetylcarnitine levels could be an important determinant of liver disease progression. It is difficult to detect non‐alcoholic fatty liver disease (NAFLD) early enough^13^ therefore the benefit of developing novel non‐invasive techniques that can detect liver metabolic flexibility is obvious.

l‐carnitine supplementation has shown to have a lipid‐lowering effect in patients with type 2 diabetes.^14^, ^15^, ^16^ Moreover, it has been observed that severe liver disease is often accompanied with low levels of tissue l‐carnitine.^17^ Therefore, as acetylcarnitine is a measure of metabolic flexibility this study aimed to investigate the hepatic metabolic flexibility by measuring the acetylcarnitine peak before and after a single intravenous supplementation with l‐carnitine in vivo using long echo‐time ^1^H‐MRS in young healthy volunteers.

## 2 METHODS

### 2.1 Phantom experiments

Phantoms were constructed to help optimize MRS acquisition parameters. All phantoms contained 5% weight/weight agar (Sigma‐Aldrich, USA), 43 mM sodium dodecyl sulfate (Sigma‐Aldrich, USA), 43 mM NaCl (Sigma‐Aldrich, USA), and 0.01% weight/volume sodium benzoate (Sigma‐Aldrich, USA). Three sets of two *N*‐acetyl‐l‐carnitine concentrations (5 mM and 10 mM, Health4All Ltd., UK) were created to reflect 0%, 5% and 10% fat fraction. Peanut oil (Sigma‐Aldrich, USA) was used as the source of triglycerides as it mimics human adipose tissue.^18^ Phantoms were stored in 30 ml plastic universal containers (King Scientific, UK).

Magnetic resonance spectra from each phantom were collected using a standard STEAM single voxel spectroscopy sequence with four TEs: TR/TE/TM = 3000/20, 100, 200, 300/20 ms, voxel size: 30 × 30 × 40 mm^3^, vector length: 1024, bandwidth 1000 Hz.

### 2.2 Subjects

Healthy volunteers were recruited through poster advertisement. The research study was approved by the National Research Ethics Committee Service (19/SC/0571), and written informed consent was obtained from all volunteers.

Fifteen healthy volunteers who were free of known disease (including a history of liver disease, diabetes, heart disease) attended for a research visit after an overnight fast. All volunteers underwent two identical 1‐h scans on the same morning. Ten volunteers received a single dose of l‐carnitine supplementation, while five volunteers served as controls and did not receive any l‐carnitine supplementation.

### 2.3 Study protocol

Following the baseline MRI scan, 10 volunteers were given an intravenous supplementation of l‐carnitine (50 mg/kg body weight, Carnitor, Alfasigma S.p.A. Italy), and 2 h after supplementation the same MRI protocol was repeated. Blood samples were acquired at baseline (T0; before imaging), and 1 h (T1), 2 h (T2), and 3 h (T3) after l‐carnitine supplementation. The five control volunteers were used to assess the effect of fasting alone on acetylcarnitine levels in the liver, hence, were imaged following the baseline scan without supplementation of l‐carnitine. No blood samples were taken from the control volunteers. Single voxel MRS was performed during each MRI scan to quantify lipid and acetylcarnitine resonances in the right lobe of the liver. Voxels were placed to avoid hepatic vasculature and bile ducts.

**FIGURE 2:**
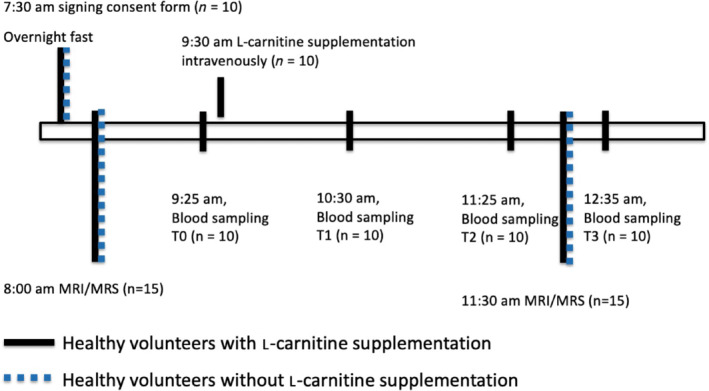
Diagram of experimental protocol. All participants were consented in the morning following an overnight fast. Livers of fifteen healthy volunteers were imaged in the morning and again 2 h later for a follow‐up scan. Ten healthy volunteers received a supplementation of l‐carnitine, and blood samples (T0, T1, T2, and T3) were collected. Five of the healthy volunteers did not receive l‐carnitine supplementation and no blood samples were acquired.

Study participants were not asked to follow a specific diet before attending the study visit. The timing of study visit elements is illustrated in Figure 2.

### 2.4 Blood samples

Blood samples were collected into tubes containing gold hemogard and then centrifuged, and the serum was immediately transferred to sterile polypropylene cryovials and submerged in liquid nitrogen before transferred to a −80°C freezer. Acetylcarnitine and free l‐carnitine was measured in serum using a clinical Tandem Mass Spectrometer protocol at the Department of Clinical Chemistry at Sheffield Children's NHS Foundation Trust and Hospital.

### 2.5 MRI and MRS protocol

#### 2.5.1 Phantoms

Spectra from phantoms were frequency aligned using water as the reference resonance using the AMARES algorithm implemented in the OXSA toolbox^19^ in Matlab (Mathworks, Natick, MA, USA). Frequency‐aligned spectra were then phase corrected and averaged. The acetylcarnitine resonance at 2.1 ppm was fitted along with lipid resonances. The SNR of the acetylcarnitine resonance at various TEs was compared for the phantoms containing 10 mM of acetylcarnitine.

#### 2.5.2 Volunteers

All measurements were undertaken on subjects lying supine in a 3T MR system (Prisma, Siemens Healthineers, Erlangen, Germany) using a 30‐channel phased array coil and a 12‐channel spine coil. The body coil of the scanner was used for excitation.

Acetylcarnitine was quantified in the right liver lobe using a free‐breathing STEAM acquisition (64 averages, TE = 100 ms [determined as optimum from previous phantom experiments], TR = 3000 ms, voxel size: 30 × 30 × 30 mm^3^). In vivo spectra were processed using the same procedure as described above for phantom spectra.

Three water‐suppressed and one non‐water‐suppressed breath‐held STEAM acquisitions were performed in the posterior part of the right liver lobe for lipid quantification. The single‐voxel STEAM sequence was synchronized to volunteers' electrocardiogram (ECG) trace (five measurements for water‐suppressed acquisitions and three measurements for non‐water‐suppressed acquisitions, TE = 10 ms, TR = 760 ms) with a pause of 2 s between measurements for water‐suppressed acquisitions, and with a pause of 4 s between measurements for non‐water‐suppressed acquisitions, voxel size: 20 × 20 × 20 mm^3^.^19^ Multiple resonances of fat were fitted; methyl (*d* = 0.9 ppm), methylene (*d* = 1.3 ppm), and allylic (*d* = 2.2 ppm) peak and were all quantified compared to the non‐supressed water signal using Equation (1), where *F* is the signal amplitude of the lipid peak and *W* is the signal amplitude of the non‐suppressed water peak.

(1) $$\begin{array}{rr} \text{Lipid peak} & =\;\frac{F\left(\text{lipid peak}\right)}{F\left(\text{lipid peak}\right)+W\left(\text{non}-\text{supressed water peak}\right)}\\ & \;\times 100\% \end{array}$$

### 2.6 Statistics

Data were reported as mean ± SD unless otherwise stated. Bland–Altman analysis was used to evaluate the agreement between the two measurements of acetylcarnitine from MRS in volunteers without l‐carnitine supplementation. The coefficient of repeatability (CR) was calculated as described in Vaz et al.^20^ Paired t‐tests were conducted to compare group means. All *p*‐values lower than 0.05 were considered significant.

## 3 RESULTS

### 3.1 Phantom results

**FIGURE 3:**
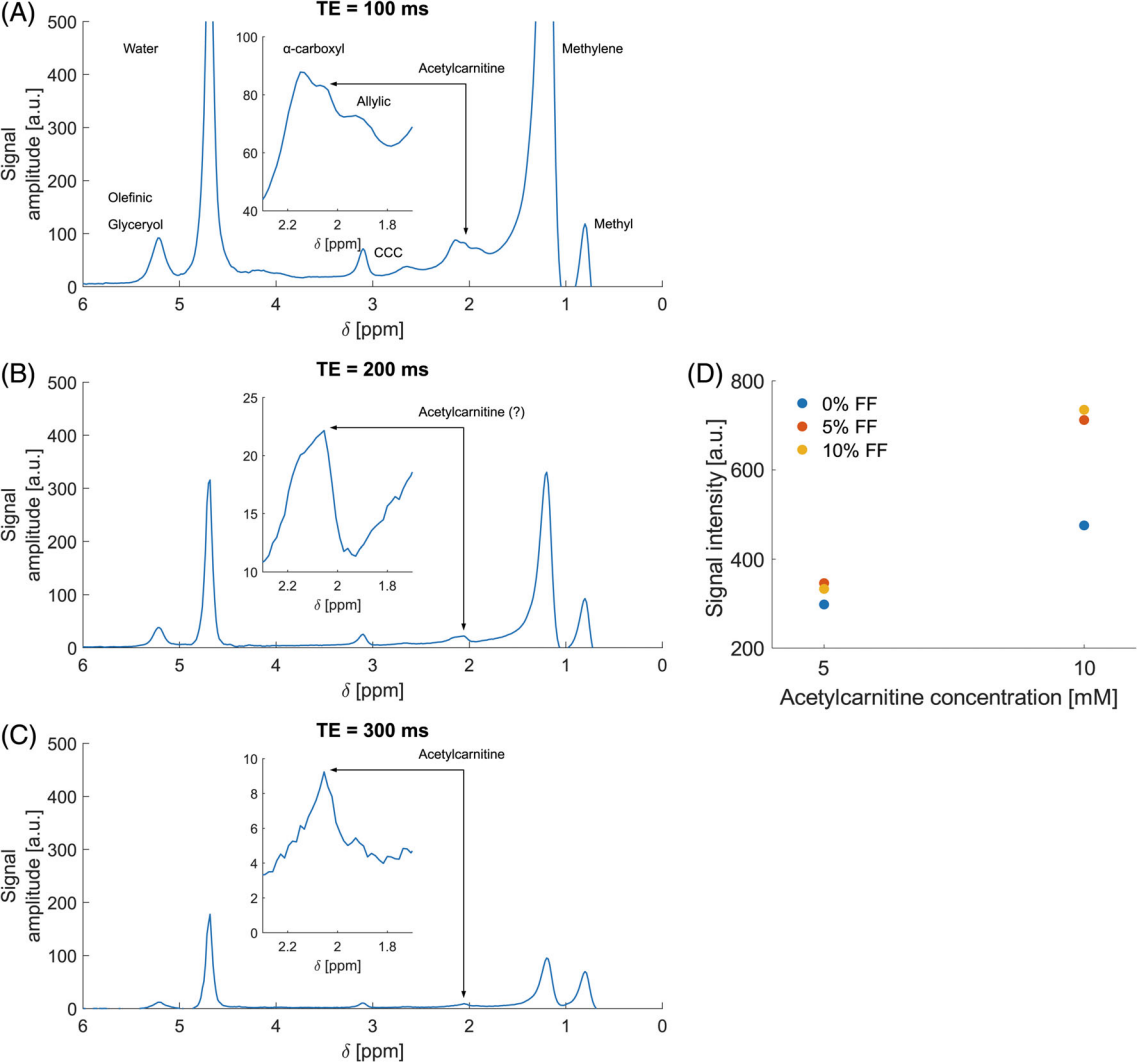
Phantom measurements. (A) Spectra acquired at TE = 100 ms. (B) Spectra acquired at TE = 200 ms, (C) Spectra acquired at TE = 300 ms. (D) Observed acetylcarnitine resonance amplitudes in phantoms grouped by theoretical concentration of acetylcarnitine and color coded by fat fraction (FF) for each phantom.

Acetylcarnitine showed good separation from the a‐olefinic resonance at TE = 100 ms, but not at TE = 200 ms, as was expected based on the J‐evolution of the a‐olefinic resonance. A visible separation was at 10% fat observed again at TE = 300 ms, but with lower SNR (SNR = 973 for TE = 100 ms; SNR = 560 for TE = 200 ms; SNR = 270 for TE = 300 ms). The acetylcarnitine resonance was easily detectable (using TE = 100 ms) at both 5% and 10% fat fraction levels at both 5 and 10 mM concentrations, as shown on Figure 3. This suggests acetylcarnitine could be detected in patients with NAFLD, as the hepatic fat present would not interfere with the quantification. Figure 3 shows spectra acquired from phantoms at various TEs.

### 3.2 Volunteers characteristics

The 10 volunteers (6 female) who received l‐carnitine supplementation, had an average age of 33 ± 9 y, and an average body mass index (BMI) of 22.7 ± 1.7 kg/m^2^. The five volunteers who did not receive l‐carnitine supplementation (one female), had an average age of 34 ± 4 y, and an average BMI of 23.7 ± 2.2 kg/m^2^.

### 3.3 Hepatic acetylcarnitine quantification

**FIGURE 4:**
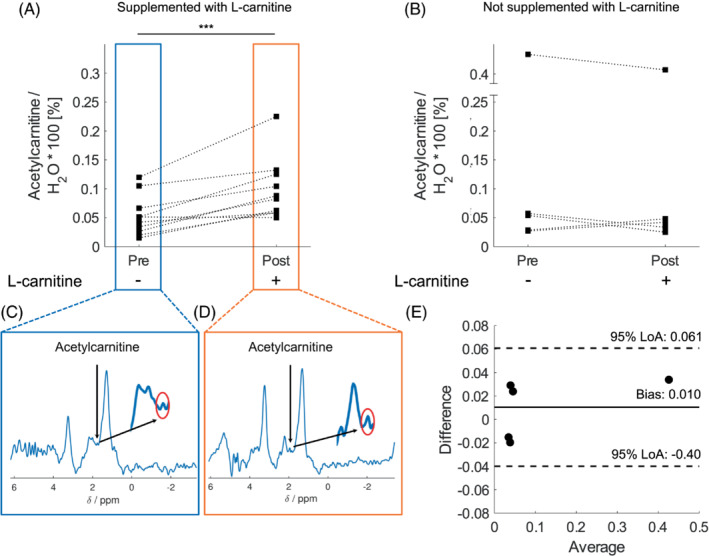
Acetylcarnitine quantified in the liver using ^1^H MRS. (A) Acetylcarnitine levels normalized to unsuppressed water signal in volunteers supplemented with l‐carnitine. (B) Acetylcarnitine levels normalized to unsuppressed water signal in volunteers not supplemented with l‐carnitine. (C) Example spectra of acetylcarnitine zoomed in on the resonance at *δ* = 2.1 ppm before l‐carnitine supplementation. (D) Acetylcarnitine MRS zoomed in on the resonance at *δ* = 2.1 ppm post l‐carnitine supplementation. (E) Bland–Altman Plot of the difference between baseline and follow up MRI of acetylcarnitine versus the average measurements at baseline and at follow up measurements from the volunteers that were not supplemented with l‐carnitine. The Bland–Altman plot shows 95% LoA = [−0.040%;0061%] and a bias = 0.010%.

Administration of a single dose of l‐carnitine elevated liver acetylcarnitine levels by 86% (*p* < 0.001, Figure 4A), while there was no change in the volunteers who did not receive a supplementation with l‐carnitine (Figure 4B). A Bland–Altman plot showed that there was good agreement (limits of agreement [LoA] = −0.040%; 0.061%), and no consistent bias (0.010%) between baseline and follow‐up scabs from the volunteers without l‐carnitine supplementation. The CR^20^ was 0.049% (Figure 4E).

### 3.4 Hepatic fat quantification

In all participants the hepatic fat fraction was below 5% in both group of volunteers and no change in the fat fraction was observed after supplementation with l‐carnitine (*p* = 0.794). Although there was one participant from the non‐supplemented group with elevated fat fraction, that increased the variability of this group. A significant change was not observed in individual lipid resonances either (Figure S1).

### 3.5 Serum acetylcarnitine measurements

**FIGURE 5:**
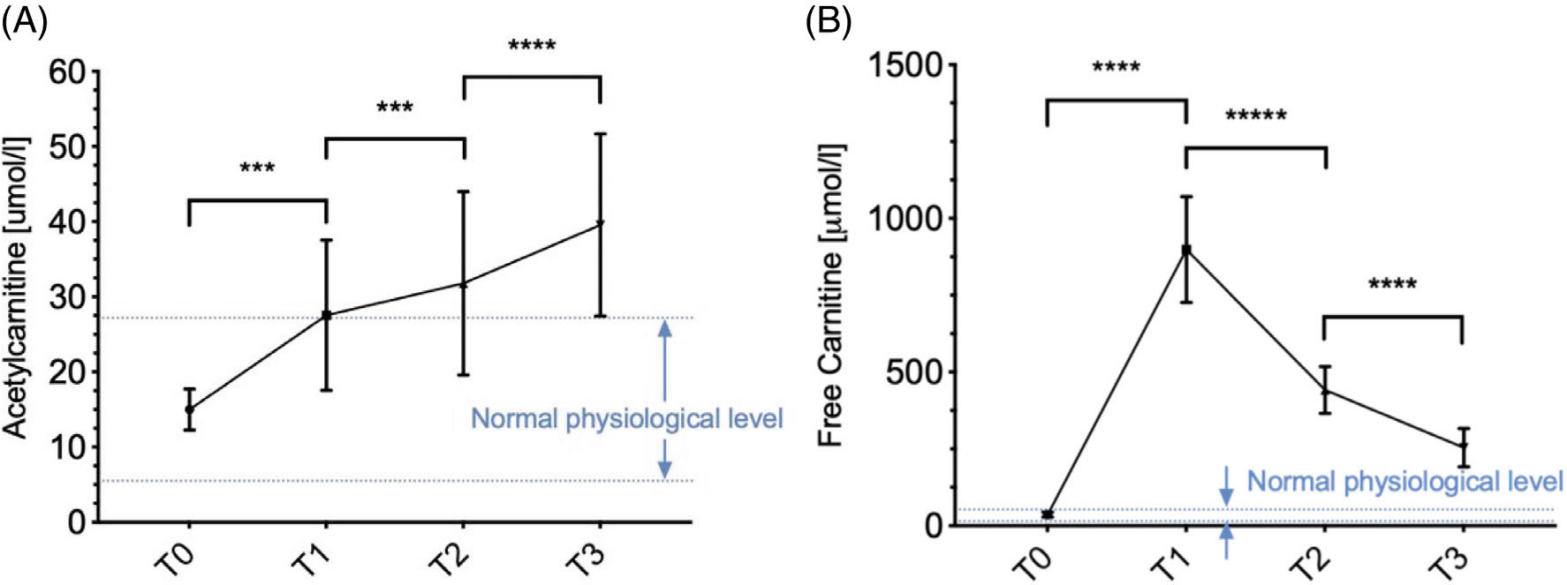
Carnitine species measured in healthy volunteer's serum from before l‐carnitine was supplemented (T0) to 1 h (T1), 2 h (T2), and 3 h (T3) after l‐carnitine was supplemented. (A) Acetylcarnitine levels; the blue dotted line represents the normal physiological values of acetylcarnitine in healthy volunteer. (B) Free l‐carnitine levels; the blue dotted line represents the normal physiological values of free L‐carnitine in healthy volunteer where ***p < 0.001, and ****p < 0.0001.

Serum acetylcarnitine levels were measured from the blood at baseline (T0) and again after l‐carnitine supplementation (T1, T2, and T3). Figure 5 shows that acetylcarnitine levels in the serum continued to increase even at the last time point (T3) which was 3 h after l‐carnitine supplementation.

## 4 DISCUSSION AND CONCLUSIONS

In this study, we have demonstrated that we can measure acetylcarnitine levels in the liver of healthy adults, using a semi‐long TE (TE = 100 ms) STEAM sequence on a 3T MRI scanner. We have also shown that administration of a single dose of l‐carnitine can modulate in vivo acetylcarnitine levels in the liver within 2 h. Thus, we now provide a platform to investigate changes in acetylcarnitine metabolism to understand why it may become impaired in some individuals, such as those with fatty liver disease.

Acetylcarnitine is a major metabolite formed during exercise and, therefore, has been studied extensively in skeletal muscle. Using a 3T MRI scanner, muscle acetylcarnitine levels were found to be lower in people with type 2 diabetes compared to healthy participants.^3^ Although acetylcarnitine levels were elevated in the blood of patients with type 2 diabetes. These blood levels more likely came from other organs, such as the liver rather than from the muscle.^21^ Plasma free carnitine were also elevated in healthy rats compared to rats with diabetes.^22^ Measured free l‐carnitine was four times higher in muscle compared to the liver^23^; however, the liver contributes more to systemic acetylcarnitine levels than muscle^12^ and, therefore, shows a need to evaluate hepatic acetylcarnitine levels in vivo.

Since the liver has lower acetylcarnitine concentration than the muscle, measuring acetylcarnitine using very long TEs comes at the cost of low SNR, which could lead to unfeasibly long scan times. Hence, we have investigated the potential to use a semi‐long TE to quantify hepatic acetylcarnitine. Song et al. reported that the J‐coupling in phantoms with different lipids is similar compared to in vivo MRS from rat livers.^24^ As expected, longer TE was equivalent to a lower intensity from several lipid peaks, however, a local minimum was observed at TE = 100 ms, which could potentially be used to suppress the lipids the most while keeping a shorter TE. There is a compromise to be made between signal‐to‐noise ratio and spectral resolution, however despite this our phantom experiments confirmed that the use of the selected sequence showed no interference from the fat peaks. We clearly observed stability in signal amplitude across the same concentration and almost a two‐fold increase in amplitude with doubled concentration of acetylcarnitine, irrespective of the fat content. The phantom study also demonstrated that the α‐olefinic resonance could be separated from the acetylcarnitine peak at TE = 100 ms, but not at TE = 200 ms as the transverse magnetization decay of fat resonances was not in a local minimum owing to J‐coupling effects (as opposed to the TE = 100 ms case). TE = 300 ms showed good separation of resonant peaks, but at the cost of lower SNR. This reduction in SNR as well as the increased localization uncertainty of the spectroscopic voxel at TE = 300 ms in human subjects made us to decide to use TE = 100 ms for in vivo experiments. This now offers an opportunity to study acetylcarnitine levels with the presence of fat peaks.

This study could not validate the acetylcarnitine levels by obtaining liver biopsies from the healthy volunteers, however by measuring acetylcarnitine levels in the serum, we have observed that l‐carnitine conversion to acetylcarnitine keeps increasing even 3 h after l‐carnitine supplementation, indicating a possible direct conversion of l‐carnitine to acetylcarnitine or an increasing release of acetylcarnitine from the liver.^22^ Of all the carnitine species, acetylcarnitine is the most common under normal physiological conditions,^6^ so after an l‐carnitine injection, this is taken up by the liver, kidneys, heart, and skeletal muscle where it is bound to acetyl‐CoA species in the mitochondria and is exported again into the blood stream.^25^ The acetylcarnitine species continue to rise for 2 h, until they are either re‐used or excreted in urine. During their study day, our participants were not using the restrooms. We were also unable to determine absolute concentration changes in hepatic acetylcarnitine; however, the absolute changes observed following the l‐carnitine supplementation would be independent of spin relaxation effects.

The repeatability experiments demonstrated that hepatic acetylcarnitine levels stay stable throughout the morning in fasted healthy adults. Although there was an outlier in the non‐supplemented group, that corresponded to the same volunteer having elevated lipid levels. Administration of a single dose of l‐carnitine resulted in increased serum acetylcarnitine levels and was accompanied by increased hepatic acetylcarnitine within 2 h. Previous studies have shown that l‐carnitine supplementation at a dose of 2 g/day for 24 wk can reduce liver inflammation, reduce plasma insulin, low density lipoprotein (LDL), and cholesterol in patients with NAFLD.^4^, ^15^ In patients with both type 2 diabetes and NAFLD who were taking l‐carnitine (500 mg/day mg for 24 wk), there was a significant reduction of liver attenuation index on computed tomography^26^ and improved sonographic grade.^27^ This could potentially mean that supplementation with l‐carnitine may reduce lipid levels in the liver. Our study only investigated acute changes post one administration of l‐carnitine and we showed no changes of the individual hepatic lipid peaks with a single‐point supplementation of l‐carnitine. It is possible that long‐term supplementation could have induced changes in individual lipid species.

We did not aim to investigate differences in l‐carnitine metabolism between male and female participants. However, there has been suggestions that l‐carnitine supplementation helps females more in post‐exercise recovery. More studies are needed to investigate potential sex‐based differences.^28^

In summary, we have demonstrated here that using a standard free‐breathing STEAM sequence with a semi‐long TE of 100 ms on a clinical 3T MRI scanner we were able to robustly detect liver acetylcarnitine levels, and that these can be modulated in vivo after a single dose supplementation of l‐carnitine. This paves the way for studies on hepatic metabolic flexibility that might prove to be useful in characterizing patients with compromised fatty acid metabolism like in diseases such as NAFLD and metabolic syndrome.

## CONFLICT OF INTEREST

MP declares no conflict of interest in relation to this study. Outside the context of this study, MP declares ownership of shares in Perspectum Ltd. LH declares no conflict of interest in relation to this study. Outside the context of the study, LH is a consultant to Novo Nordisk. MK is full time employee and shareholder of Novo Nordisk A/S.

## Supporting information


**Figure S1.** Lipid ^1^H MRS acquired from the liver. Healthy volunteers with l‐carnitine supplementation (illustratedas hollow bar graphs) and healthy volunteers without l‐carnitine supplementation (illustrated with striped bar graphs). (a) Hepatic allylic fat peak normalized to unsuppressed water. (b) Illustrative image of the liver with showing positioning of the voxel used to quantify lipids. (c) Hepatic methyl fat peak normalized to unsuppressed water. (d) Hepatic methylene fat peak normalized to unsuppressed water. Standardized paired *t*‐test between baseline and post 2 h of l‐carnitine/without l‐carnitine supplementation showed no significant changes. The box plots depict medians and interquartile ranges; the whiskers are extending to the maximum and minimum value.
