# Supplementary material for: Detection and alterations of acetylcarnitine (AC) in human liver by 1H MRS at 3T after supplementation with l‐carnitine
Source: Magn Reson Med. 2022 Dec 27;89(4):1314–22. doi: 10.1002/mrm.29544 (PMC11497247; doi:10.1002/mrm.29544)
Supplement: Supplementary file 1 — Figure S1. Lipid 1H MRS acquired from the liver. Healthy volunteers with l‐carnitine supplementation (illustratedas hollow bar graphs) and healthy volunteers without l‐carnitine supplementation (illustrated with striped bar graphs). (a) Hepatic allylic fat peak normalized to unsuppressed water. (b) Illustrative image of the liver with showing positioning of the voxel used to quantify lipids. (c) Hepatic methyl fat peak normalized to unsuppressed water. (d) Hepatic methylene fat peak normalized to unsuppressed water. Standardized paired t‐test between baseline and post 2 h of l‐carnitine/without l‐carnitine supplementation showed no significant changes. The box plots depict medians and interquartile ranges; the whiskers are extending to the maximum and minimum value. [file MRM-89-1314-s001.docx]

Supporting Information for Detection and alterations of acetylcarnitine in human liver by ^1^H MRS at 3T after supplementation with L-carnitine


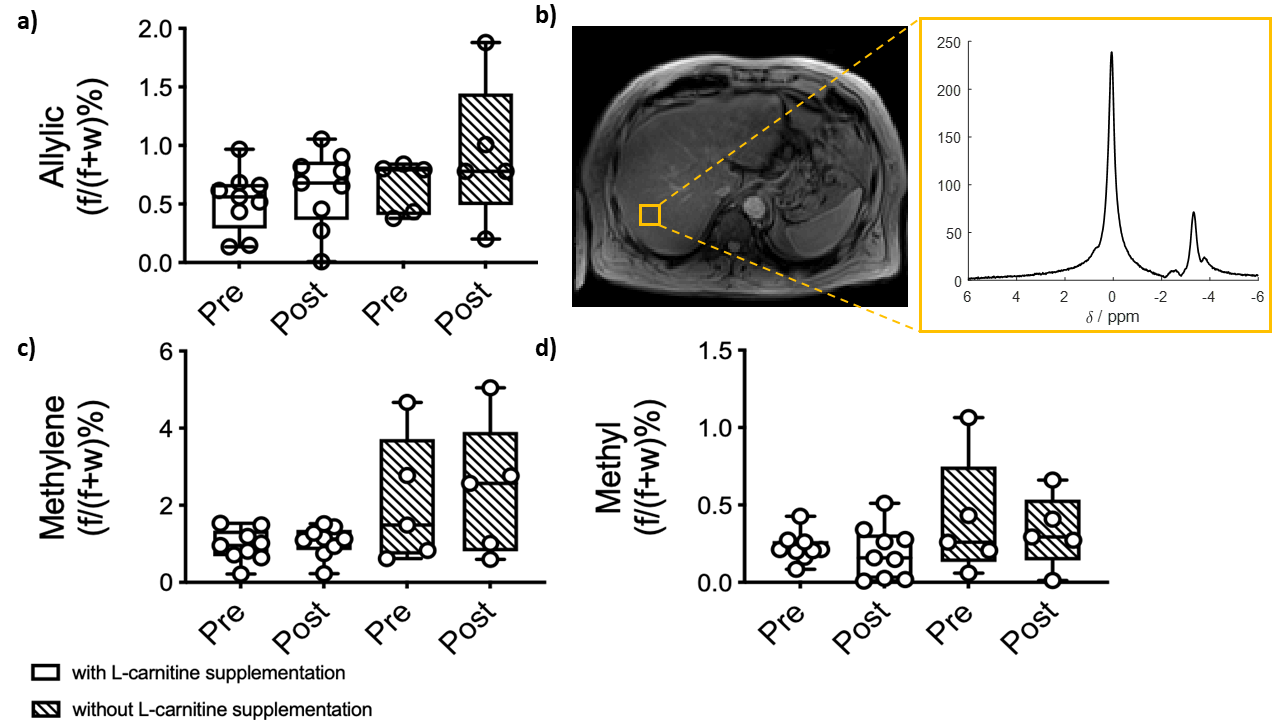


**Supporting Figure 1** Lipid ^1^H MRS acquired from the liver. Healthy volunteers with L-carnitine supplementation (illustrated as hollow bar graphs) and healthy volunteers without L-carnitine supplementation (illustrated with striped bar graphs. a) Hepatic allylic fat peak normalized to unsuppressed water. b) Illustrative image of the liver with showing positioning of the voxel used to quantify lipids. c) Hepatic methyl fat peak normalized to unsuppressed water. d) Hepatic methylene fat peak normalized to unsuppressed water. Standardized paired t-test between baseline and post 2 hours of L-carnitine/without L-carnitine supplementation showed no significant changes. The box plots depict medians and interquartile ranges; the whiskers are extending to the maximum and minimum value.
